# Quantifying Experimental Variability in Shear-Induced Hemolysis to Support Uncertainty-Aware Hemolysis Models

**DOI:** 10.1007/s10439-025-03786-z

**Published:** 2025-07-04

**Authors:** Christopher Blum, Markus Mous, Ulrich Steinseifer, Johanna C. Clauser, Michael Neidlin

**Affiliations:** Department of Cardiovascular Engineering, Institute of Applied Medical Engineering, Medical Faculty, RWTH Aachen University, Aachen, Germany

**Keywords:** Experimental hemolysis, Inter-variability, Intra-variability, Uncertainty quantification

## Abstract

**Purpose:**

Numerical hemolysis models rely on experimental data to fit parameters and predict hemolysis under various conditions. However, existing experiments often use few replicates per condition, leaving inherent variability largely unaddressed. This can lead to oversimplified models that fail to capture the true nature of hemolysis. Here, we quantify intra- and inter-donor variability at a single, well-defined shear stress and exposure time and examine how sample size affects measurement precision

**Methods:**

Human blood from five healthy donors was subjected to a fixed shear stress and exposure time condition. For each donor, 20 independent measurements were performed to calculate a hemolysis index (HI). Intra-donor variability (variation within a single donor’s measurements) and inter-donor variability (variation between donor means) were compared. Additionally, bootstrap analyses were used to explore the effect of the sample size on the confidence intervals of the mean HI.

**Results:**

Intra-donor variability was approximately four times higher than inter-donor variability, indicating that most of the uncertainty originated from within a single donor’s set of samples rather than between donors. Increasing the sample size from 2 to 20 replicates substantially narrowed the confidence intervals of the mean hemolysis estimate, suggesting that commonly used small sample sizes may underrepresent the true variability in hemolysis measurements.

**Conclusion:**

Intra-donor variability is a significant driver of uncertainty in hemolysis measurements at a fixed shear stress and exposure time condition, surpassing differences among donors. Obtaining robust and reliable hemolysis estimates requires increasing the number of replicate measurements to reduce uncertainty. Integrating these insights into future experimental designs and uncertainty-aware hemolysis models will improve the reliability of in silico predictions and inform safer, more effective blood-contacting medical device designs.

## Introduction

Hemolysis, the release of hemoglobin from red blood cells into the plasma, poses significant challenges in the use of blood-contacting devices that subject red blood cells to supraphysiological shear rates. While relevant in various applications such as mechanical heart valves [1] and ventricular assist devices (VADs) [2], hemolysis is particularly critical in extracorporeal membrane oxygenation (ECMO) systems. In ECMO, elevated levels of hemolysis have been linked to increased patient mortality [3] and severe complications, including thrombosis and bleeding events [4]. Consequently, reducing hemolysis by informed design choices remains a priority for improving the safety and efficacy of these life-saving technologies.

To address this issue, in silico modeling has emerged as a valuable tool for device optimization. By allowing rapid testing and refinement of design choices, computational approaches reduce the need for extensive in vitro or in vivo experimentation [5]. Reflecting the growing importance of computational methods, regulatory authorities such as the U.S. Food and Drug Administration (FDA) have emphasized verification, validation, and uncertainty quantification (VVUQ) as essential elements for approving blood-contacting medical devices [6]. Central to the credibility of in silico hemolysis models is the experimental data on which they are based.

To inform numerical hemolysis models, various experimental strategies have been pursued. At the microscopic scale, several studies focused on probing the structural membrane properties of individual RBCs and assessing the correlation between applied stress and membrane rupture [7–9]. While these analyses offer detailed insights into cellular-level mechanics, translating these findings into large-scale flows typical of cardiovascular devices, and subsequently incorporating them into continuum-based hemolysis models, is a complex and challenging task. Moreover, experiments have been conducted to capture the influence of different flow conditions (laminar versus turbulent) [10] and to determine threshold values beyond which hemolysis occurs [11–13] . However, these measurements can strongly depend on the specific experimental setup and the prevailing flow characteristics, which limits their generalizability and complicates direct comparisons across studies.

A more commonly adopted approach is to expose whole blood to controlled pure shear stresses for defined exposure times using specialized shearing devices to measure a hemolysis signal under a range of conditions. Since the pioneering work of Giersiepen et al. [14], numerous experiments have been conducted for different species [15] and various experimental setups [16–18]. From these datasets, empirical Power Law relationships between shear stress, exposure time, and hemolysis have been derived. Such relationships are straightforward to integrate into numerical models. Despite these efforts, no universal set of Power Law fitting parameters reliably describes the shear stress, exposure time, and hemolysis relationship across all experimental conditions. Fitting coefficients vary widely between studies, possibly reflecting differences in experimental methodologies, sample preparation or measurement techniques. Due to constraints on time and resources, most studies aim to investigate a wide range of conditions with limited replicates per condition (often just three). For instance, Zhang et al. [18] examined shear stresses ranging from 30 to 330 Pa and exposure times of 0.03 to 1.5 s across 42 conditions, each tested in triplicate. With mean coefficient of variation of the triplicate measurements for all conditions being approximately 30%, indicating not only fitting parameter variability across different studies but also substantial within-study variability, making it challenging to identify universally valid fitting parameters.

Although these foundational studies have supported the use of numerical hemolysis modeling as a valuable tool for comparative device optimization, large discrepancies in predicted outcomes remain a significant limitation when absolute hemolysis values are desired. Taskin et al. demonstrated that predicted hemolysis can differ by several orders of magnitude depending on the modeling approach (Eulerian vs. Lagrangian) and the chosen fitting coefficients [19]. Similarly, Yu et al. [20] noted that the selection of fitting parameters is the primary source of deviation between model predictions and actual measurements. Beyond uncertainties in parameter selection, recent studies have also raised conceptual concerns with current hemolysis models, such as the treatment of exposure time in accelerating flows and the use of simplified representations of shear stress [21, 22]. Moreover, the simplest forms of the Power Law equation may not yield unique solutions, as highlighted by Craven et al. [23].

These challenges indicate that both experimental and modeling limitations continue to hinder the accurate prediction of absolute hemolysis values. From an experimental perspective, the root cause of this challenge may lie in the substantial uncertainty embedded in experimental data. The conventional approach of fitting a single curve to mean values neglects inherent variability, potentially masking the range of plausible outcomes and leading to models that underestimate uncertainty. As a result, identifying truly representative model parameters may be unfeasible with current methodologies.

In previous work [24], we introduced a Bayesian parameter inference framework employing Markov-Chain Monte Carlo methods to incorporate experimental variability into hemolysis models. While this approach can capture uncertainty, it still relies on published datasets with relatively small sample sizes for each shear stress and exposure time condition. Thus, a robust quantification of experimental uncertainty in hemolysis measurements remains unachieved, and further investigation is needed to pinpoint the sources of this variability. Addressing this gap requires a more detailed examination of variability within the experimental data. Instead of mapping hemolysis across many shear stress and exposure time conditions to gain insights about the functional relationship between these two factors, this study focuses on a single, well-defined shear stress and exposure time condition. By dedicating a substantial number of replicates (n = 100) to a single scenario, rather than spreading resources across multiple conditions, we can thoroughly investigate both intra-donor and inter-donor variability. This approach enables us to discern which source of variability is more critical and how the number of replicates (sample size) influences the precision of hemolysis estimates.

In doing so, we aim to answer two key questions: (1) Is the observed variability primarily driven by intra-donor differences or inter-donor differences? (2) How does sample size affect the observed variability and the reliability of mean hemolysis estimates? Ultimately, these insights will pave the way for more robust and uncertainty-aware hemolysis models. By better characterizing the variability in experimental data, future in silico predictions can more accurately reflect the real range of outcomes, informing device developers, guiding regulatory decisions, and contributing to safer, more effective blood-contacting devices.

## Materials and Methods

To investigate the intra- and inter-experimental variability of hemolysis measurements, we developed a high-precision shearing device based on a rotational rheometer. Using this setup, we conducted n = 5 experiments with human blood obtained from healthy donors approved by the local Institutional Review Board of the University Hospital Aachen (approval number 23-199). For preliminary tests, porcine abattoir blood was used with well-established acquisition protocols [25].

### Test Setup

Rotational rheometers are well-established instruments known for their ability to generate controlled and well-defined flow conditions. They provide exceptional measurement precision, stable temperature control, and superior reproducibility. In this study, we employed a rotational rheometer (502e, Anton Paar, Austria) with a custom-made rotational bob, capable of a maximum rotational speed of 3000 rpm.

Our primary objective was to create reproducible, supraphysiological shear stress conditions inside the rheometer. This required an optimal balance between gap size, sample volume, geometry diameter, and manufacturing tolerances. The resulting configuration was a Mooney-Ewart shearing geometry, which is essentially a combination of cylinder-cylinder and cone-plate designs. This geometry enabled us to maximize the proportion of sample subjected to a uniform shear rate, ensuring that 98.5% of a 180 μL blood sample experienced a consistent shear rate of approximately 45,000 s⁻^1^. Assuming a dynamic viscosity of 3.5 mPa s, the corresponding shear stress was approximately 160 Pa, mirroring mechanical circulatory support conditions [26, 27]. The sample volume of 180 μL was sufficient to perform duplicate hemolysis measurements.

**Fig. 1:**
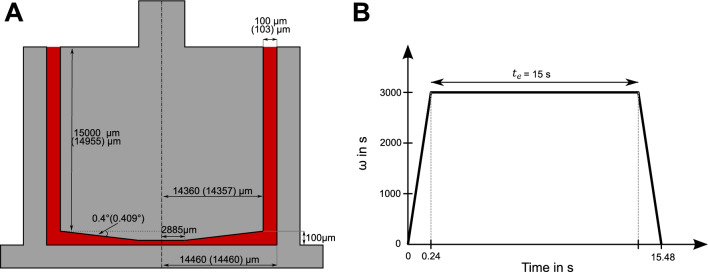
Schematic representation of the Mooney-Ewart shearing geometry (**A**), featuring a rotating inner bob and a static outer cylinder (gray). The blood sample volume is illustrated in red. Nominal dimensions of the geometry are provided, with the actual measured dimensions after manufacturing shown in parentheses. The acceleration and deceleration profile of the measurement system is depicted in (**B**), illustrating the rotational speed (ω) ramping up to 3000 rpm. The period of constant rotational speed, lasting 15 seconds, is defined as the exposure time

Figure 1A provides a schematic of the Mooney-Ewart geometry, with a nominal gap size of 100 μm and a nominal cone angle of 0.4°. The inner and outer cylinders were machined in a single process, ensuring a concentricity of ± 3 μm under operating conditions, including the maximum runout of 1 μm of the rheometer’s air-bearing coupling, as certified by the manufacturer. The measured dimensions yielded an actual gap size of 103 μm, corresponding to a maximum shear rate of approximately 43,600 s⁻^1^ at 3000 rpm. Given the system’s moment of inertia, the rheometer can accelerate to 3000 rpm in 0.24 s and decelerate to zero in the same time interval.

To assess flow stability at maximum speed, we calculated the Reynolds number (~170) and the Taylor number (~0.0095). Both values are well below their respective critical thresholds for the onset of flow instabilities. In particular, the Taylor number is several orders of magnitude below the critical value of 1708, confirming that the flow remains laminar. Under these conditions, shear is applied uniformly across the narrow gap without the formation of secondary flow structures such as Taylor vortices.

In this study, the exposure time (${t}_{e}$) was defined as the duration during which the rheometer maintains a constant rotational speed, as illustrated in Figure 1B. This choice was informed by preliminary experiments conducted with porcine blood, where exposure times ranging from 0.5 to 25 seconds were tested. From previous literature [15, 18], we knew that increasing shear stress and exposure time tends to raise both the hemolysis index (HI) and its variability. To allocate experimental resources toward a single, well-defined operating point for assessing intra- and inter-donor variability, we selected an exposure time of 15 seconds that reliably produces measurable hemolysis while maintaining reproducibility. To achieve a target HI of approximately 0.5–1.0%, which corresponds to the high HI range of previous literature, we selected an exposure time of 15 seconds based on an experiment with porcine blood at hematocrit of 30% (Supplemental Information, Figure SI 1). This decision accounted for the reduced hematocrit of 25% in our proposed experimental setup with the corresponding decrease in viscosity and shear stress and the greater shear resistance of porcine blood compared to human blood [28]. Additional preliminary tests with porcine blood, involving only acceleration and deceleration phases without a steady shear period, showed no significant increase in hemolysis (n = 6), confirming our assumption that hemolysis happens only during the plateau phase of the shear rate profile.

### Blood Collection and Handling

Human blood was collected from healthy volunteers via venipuncture into 9-mL ethylenediaminetetraacetic acid (EDTA) tubes (S-Monovette K2E Gel, Sarstedt, Germany) to ensure cell stability and allow for accurate blood counts, as recommended by the International Council for Standardization in Hematology [29]. After 10 minutes of gentle mixing, the blood was centrifuged at 5000 g for 15 minutes to produce platelet-poor plasma (PPP). This PPP was then recombined with whole blood at the appropriate ratio to achieve a hematocrit of 25 ± 1%, reflecting values relevant to ECMO conditions adhering to best clinical practice with a transfusion trigger of 7 g/dL hemoglobin [30].

Following hematocrit adjustment, the blood was transferred using an automated pipette (Xplorer Plus, Eppendorf, Germany) at the lowest speed setting with 1000 μL tips having a minimal opening diameter of 0.77 mm. This approach minimized wall shear stress (<10 Pa, assuming a viscosity of 3.5 mPa s) and allowed for maximal consistency in blood handling. The blood was then transferred into 1.5-mL microtubes (Safe-Lock Tube, Eppendorf, Germany) and mounted in a custom 3D-printed cylindrical holder. This assembly was gently rotated at 10 rpm (BTR5-12 V tube roller mixer, Ratek, Australia) throughout the experiment day to ensure uniform mixing. 

Before and during the experiments, blood samples were routinely analyzed for standard red blood cell metrics (ProCyte Dx, Idexx, USA) as well as pH, lactate, and glucose levels (ABL 825 Flex blood gas analyzer, Radiometer, Germany). All sampling and handling steps were performed under identical conditions to ensure maximal consistency and comparability across experiments.

### Experimental Protocol and Cleaning Procedure

All experiments were conducted at 37 °C to replicate in vivo viscosity conditions. Viscosity measurements, taken after dilution to a hematocrit of 25 ± 1%, were performed at the start and end of each experimental day, using the same rotational rheometer. Across all five donors, the viscosity at a shear rate of 1000 s⁻^1^ had a mean value of 2.9 mPa s with a standard deviation of 0.09 mPa s. Although direct measurements at the experimental shear rate (~45,000 s⁻^1^) were not feasible, blood is known to exhibit only minor changes in viscosity at shear rates above 1000 s⁻^1^. Therefore, the value measured at 1000 s⁻^1^ is considered representative for the conditions applied in our experiments.

For each experimental run, blood from a single 1.5-mL microtube was divided into two samples: a zero sample (180 μL) transferred into a 200-μL microtube and a rheometer sample (180 μL) subjected to 15 s of shear at 3000 rpm in the rheometer.

Before each new measurement, the rheometer geometry was cleaned with deionized water to ensure osmotic hemolysis eliminated any residual red blood cells. After flushing with deionized water, the system was rinsed with NaCl solution (B. Braun, Germany) and dried with purified, pressurized air. The geometry was then reheated to 37 °C and allowed to equilibrate for 10 minutes.

The relative vertical distance between the rotating and static parts of the Mooney–Ewart shearing geometry was redefined before each measurement by determining the zero-gap position at a force threshold of 1 N. Subsequently, 180 μL of blood was placed at the center of the outer cylinder’s bottom surface, using reverse pipetting to ensure bubble-free application. The inner cylinder then approached the measurement gap at a controlled rate, starting at 8000 μm/s and slowing progressively to 1 μm/s near the final position. This procedure minimized pre-shearing and ensured even blood distribution within the gap. Once the gap was set, the temperature was confirmed to remain within ± 0.1 °C for at least 20 seconds before initiating shearing.

After the shearing step, the inner cylinder was retracted following a similar speed profile, starting slowly (1 μm/s) to minimize additional shear before increasing speed once the gap geometry no longer influenced the blood sample. The sheared blood was then transferred to a 200-μL microtube and, together with the zero sample, centrifuged at 2000 g for 15 minutes. The separated plasma was centrifuged again (2000 g, 15 minutes) to ensure removal of any residual cellular components, and then stored at − 30 °C until subsequent hemolysis analysis.

### Analysis of Hemolysis

Hemolysis was quantified in accordance with DIN 58931:2021-09 using the cyanmethemoglobin (HiCN) method. Plasma samples were thawed in a 37 °C water bath for 8 minutes and diluted 1:5 (v/v) with HiCN conversion solution (fHb (HiCN), Bioanalytic GmbH, Germany). The dilutions were prepared in duplicate on a 96-well microplate (96 Well Half Area Microplate, Greiner Bio-One, Austria). Following a 30-minute incubation, plasma-free hemoglobin (pfHb) was measured photometrically at 540 nm, with a reference wavelength of 680 nm, using a microplate reader (Spark Multimode Microplate Reader, Tecan, Switzerland). All readings were blank-corrected and converted to mg/dL pfHb following Tapernon et al. [31] . Quality controls at concentrations of 12.5, 39, 50, and 250 mg/dL pfHb were included on each plate to confirm measurement validity and calibration range. Each sample was measured in duplicates, and the mean value of the duplicates was used for subsequent analyses.

To facilitate direct comparisons with literature values, the previously described hemolysis index (HI), as defined in equation 1, was calculated.

1 $$HI = \frac{{\Delta pfHb*\left( {100 - HCT} \right)}}{Hb}$$

First, a ΔpfHb (delta plasma-free hemoglobin) value was determined by subtracting the pfHb level of the zero sample from the pfHb level of the sheared sample (ΔpfHb = pfHb_sheared – pfHb_zero). Importantly, both the sheared and zero samples were derived from the same 1.5-mL microtube, ensuring they shared the same blood handling and time history, thereby minimizing variability between paired measurements. This ΔpfHb was then adjusted for the individual hematocrit (HCT) and hemoglobin (Hb) values, which varied slightly between donors (see Table 1 and Figure SI2). Mean HCT and Hb values from the entire experimental day were used for the adjustment. The resulting HI thus provides a normalized measure of hemolysis that accounts for variations in blood composition, enabling a more meaningful comparison to established literature standards.

**Table 1:** Baseline mean values and standard deviations across all 5 donors, including demographics (age, sex, and body mass index (BMI)), selected hematological red blood cell parameters (hematocrit (HCT), total hemoglobin (Hb), mean corpuscular volume (MCV), mean corpuscular hemoglobin concentration (MCHC), and red cell distribution width standard deviation (RDW-SD)) measured before hematocrit adjustment, and additional metabolic parameters (pH, glucose, and lactate) measured after hematocrit adjustment

	Parameter	Mean	Standard deviation
Demographics	Age/(years)	36.8	11.12
	Sex/(% female)	40	N/A
	BMI/(kg/m^2)	24.01	2.32
Before hematocrit adjustment	Hematocrit/(%)	39.72	2.27
	Total hemoglobin/(g/dL)	14.32	0.95
	MCV/(fL)	86.62	3.36
	MCHC/(g/dL)	36.08	0.85
	RDW-SD/(fL)	41.28	2.87
After hematocrit adjustment	Hematocrit/(%)	24.54	0.35
	Total hemoglobin/(g/dL)	8.92	0.16
	MCV/(fL)	85	3.39
	MCHC/(g/dL)	36.36	1.05
	RDW-SD/(fL)	40.04	3.02
	pH/(-)	7.28	0.04
	Glucose/(mmol/L)	5.02	0.74
	Lactate/(mmol/L)	1.94	0.42

### Statistical Analysis

All statistical analyses were performed using Python (version 3.11.7) and standard scientific computing libraries, including NumPy for numerical computations [32], pandas for data handling [33], and Matplotlib for data visualization [34].

To assess whether variability in our measurements was primarily driven by inter-donor variability or intra-donor variability, we performed a variance components analysis. This method allows the total variability in the data to be decomposed into these distinct sources. For this each measurement, Y_ij_ was modeled using equation (2):

2 $$Y_{ij} = \mu + d_{i} + \varepsilon_{ij},$$

where $\upmu$ is the overall mean, ${d}_{i}$ is the donor-specific effect (with variance ${\tau }^{2}$), and ${\varepsilon }_{ij}$ is the intra-donor measurement noise (with variance ${\sigma }^{2}$).

To quantify the proportion of variance attributable to donor differences, we calculated the intraclass correlation coefficient (ICC) according to in equation (3):

3 $${\text{ICC}} = \frac{{\tau^{2} }}{{\tau^{2} + \sigma^{2} }}$$

To quantify uncertainty and to assess how the number of replicate measurements per donor affects the estimated mean HI, a bootstrap analysis was conducted. Bootstrapping is a non-parametric resampling technique that does not assume an underlying distribution of the data. In this study, the bootstrap samples were generated with replacement, ensuring that each resampled dataset could be selected multiple times. Each bootstrap run consisted of 10,000 resampling iterations, and the 95% confidence intervals were determined by taking the 2.5th and 97.5th percentiles of the resulting bootstrap distribution.

## Results

### Baseline Characteristics of Blood Samples

Table 1 summarizes the mean and standard deviation of donor demographics, standard red blood cell metrics, and metabolic parameters (pH, glucose, lactate) for all five donors before and after dilution to a hematocrit of 25 ± 1%.

All donors exhibited values within normal physiological ranges. Temporal progression of these parameters over the entire experimental day is provided in Supplementary Information Figure SI2.

### Hemolysis Index (HI) Variability Among Donors and Experiments

**Fig. 2:**
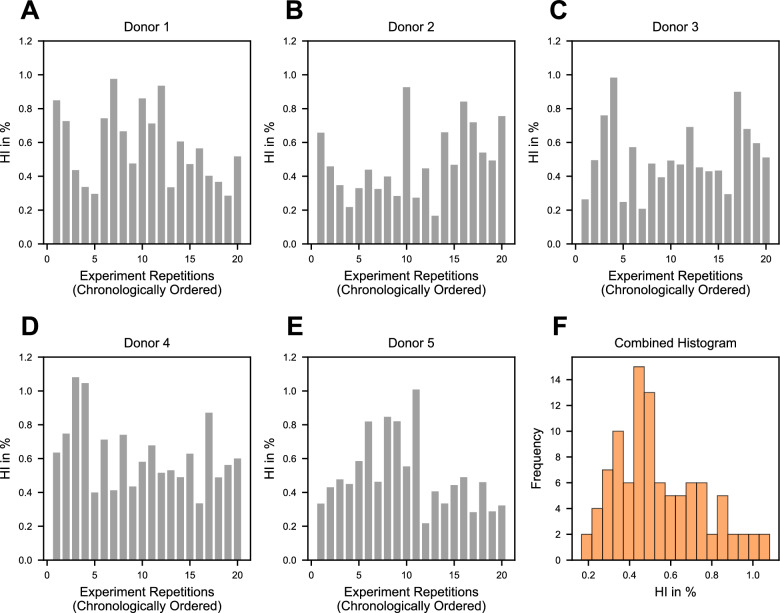
Chronologically ordered hemolysis index (HI) measurements for each donor are shown as bar charts in panels **A**–**E**, with 20 individual gray bars numbered 1–20 for each donor. Panel F displays the combined histogram of all HI measurements across all donors, represented in orange

Figure 2A–E presents the hemolysis index results for each of the five donors across 20 consecutive experimental runs. Within each donor, individual hemolysis measurements ranged approximately from 0.2% to 1.1% HI, with a similar degree of variation observed across all donors.

No clear temporal trend was observed over the course of the 20 runs. The combined distribution of all 100 measurements (20 runs × 5 donors) is shown in Figure 2F, indicating a positively skewed distribution with a mean HI near 0.5%.

### Intra- and Inter-Donor Variability

**Fig. 3:**
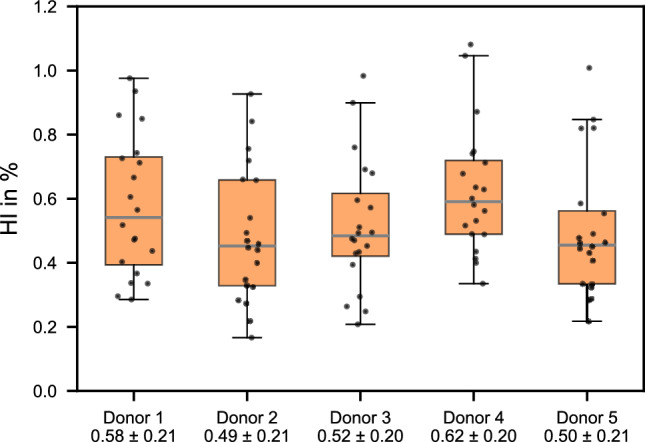
Hemolysis index (HI) measurements in percentage for each donor, displayed as box plots. The lower and upper edges of each box represent the 25th and 75th percentiles, respectively, while the whiskers indicate the 95th percentile. The mean value is shown as a horizontal line within the box and is also provided numerically along with the standard deviation in the description of the abscissa for each donor. Individual measurements are represented by scatter points overlaid on the box plots

Figure 3 shows a boxplot comparing the distribution of HI values for each donor and the variation of the HI measurements between the donors. The means and standard deviations for each donor are provided at the bottom of Figure 3.

As has already been observed in Figure 2, there is a similar degree of variation for each donor with similar means and standard deviations. However, the variation within each donor’s measurements (intra-donor variability) is higher than the variation between donor means (inter-donor variability). A quantification of the variation by computing the coefficient of variation (CV) is shown in Table 2 that presents various potential sources of uncertainty.

**Table 2:** Coefficient of variation (CV) in percentage (%) for various potential sources of variation, including intra-variability, inter-variability, measurement variability, and photometer measurement variability

	CV in %
Intra-variability (average over 5 donors)	39.3
Inter-variability (across donor means)	9.5
Measurement variability (average over 100 duplicates)	2.2
Photometer accuracy (triplicate measurement)	0.08

Further analysis (Table 2) revealed an intra-donor CV of 39.3% and an inter-donor CV of 9.5%. Additional sources of variability that could be quantified included the measurement variability across duplicate measurements (2.2%) and the photometric readout variability (0.08%), determined by repeated absorbance measurements of the same well plate using the microplate reader. When disregarding minor variability sources, the ratio of intra- to inter-donor CV was approximately 4.1.

Applying the variance components analysis to our measurements led to ${\sigma }^{2}$ = 0.044424 and ${\tau }^{2}$ = 0.001116, yielding an intraclass correlation coefficient of 0.0245. This indicates that only 2.45 percent of the total variance can be attributed to inter-donor differences, while the remaining 97.55 percent is due to intra-donor variability.

### Impact of Sampling Frequency on HI Mean Estimates

**Fig. 4:**
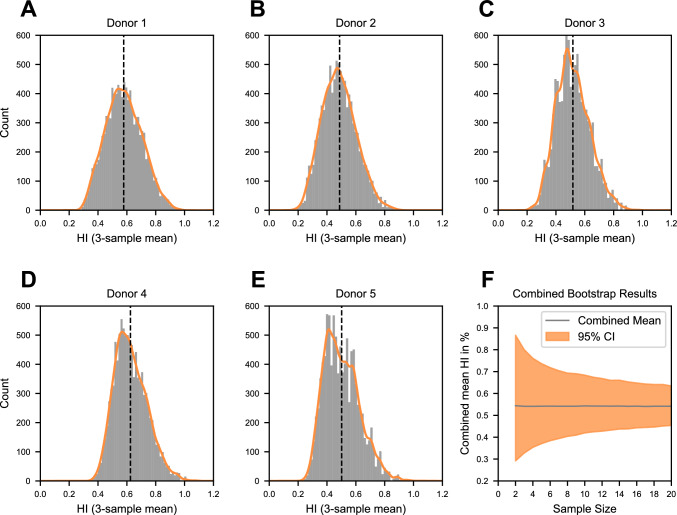
Bootstrapped distributions of possible mean values for a theoretical sample size of 3 for each donor are shown in panels **A**–**E**. The distributions are represented by gray histograms and approximated using a Gaussian kernel density estimate (orange). The black dashed line indicates the mean value for each donor based on the original 20 samples measured in this study. Panel F displays the bootstrapped confidence interval progression across sample sizes ranging from 2 to 20 for all combined samples (orange), with the total mean of all donors represented as a gray line

To assess how the number of replicate measurements per donor affects the estimated mean HI, a bootstrap analysis was conducted. Figure 4 A–E shows the distributions of bootstrapped mean HI values when randomly selecting 3 out of the 20 measurements per donor.

Across donors, mean values derived from subsets of measurements ranged from approximately 0.2% to 1.0% HI, with the distribution centers aligning closely with the overall mean of all 20 measurements (dashed black lines). In other words, if experiments were conducted with only three measurements per donor, the inherent variability could result in selecting three outliers by chance. This would lead to a mean HI value at the edges of the distribution, such as 0.2% or 1.0%, highlighting the risk of underestimating or overestimating the true mean due to limited sampling. Figure 4F displays the possible variation in mean HI as a function of sample size (2 to 20). With only two measurements, the 95% confidence interval (CI) around the mean was ± 0.3% HI. Increasing the sample size to five measurements narrowed the CI to ± 0.2% and increasing it further to twenty measurements reduced the CI to ± 0.1%. This pattern demonstrates an asymptotic decrease in variability as the sample size grows. For illustration, increasing the sample size from twenty to a theoretical one hundred measurements (requiring eighty additional samples) would reduce the CI to ± 0.05% HI.

## Discussion

This study aimed to dissect the variability inherent in hemolysis measurements under a single, well-defined shear stress condition and exposure time, distinguishing between intra-donor and inter-donor variability. By conducting a large number of experiments (20 replicates per donor, 5 donors, totaling 100 measurements) under identical conditions, we not only quantified the mean level of hemolysis but also the underlying variability. These insights have important implications for future experimental designs and for incorporating uncertainty into numerical hemolysis models. We hypothesized that using a low sample number in experimental hemolysis measurements might obscure the results due to a large intra-subject variation.

Our results show that intra-donor variability is approximately four times higher than inter-donor variability. This challenges the commonly held assumption that donor-to-donor differences [35] or inter-laboratory handling protocols [36] are the primary drivers of variability. Despite all donors being healthy, having similar physiological baseline parameters and undergoing identical sample handling procedures, the range of hemolysis index values within a single donor exceeded the variation observed between donor averages. Consequently, the expected donor-specific differences may be overshadowed by these intra-donor factors.

One potential explanation for this inherent stochasticity observed across all donors could be red blood cell fragility [20, 37]. Such stochasticity may be further amplified by the small sample volume of 180 μL, where the composition of red blood cells or the influence of outliers may have a disproportionately large effect. Each of the 180 μL samples in our study contained approximately 500 million RBCs. A hemolysis index of 1% corresponds to the release of hemoglobin equivalent to the content of roughly 2 million red blood cells. This estimate assumes complete rupture of the affected cells, without accounting for potential pre-lytic hemoglobin release through transient pores or membrane thinning. Given this assumption and the sensitivity of the measurement, our analysis (Supplementary Figure S3) suggests that even subtle variations in measured cell volume distribution across samples may contribute to intra-donor differences in HI measurements of approximately ±0.1%. While this exploratory analysis does not fully account for the total observed intra-donor variability, it provides a plausible explanation and identifies a potential direction for future studies aimed at investigating the origins of variability in hemolysis measurements.

It should also be noted that despite substantial effort and resources invested in developing a highly precise shearing geometry, absolute accuracy and reproducibility cannot realistically be attained in practice. Given that only a small fraction of RBCs is needed to rupture to influence the results, small deviations in operating tolerances such as micrometer-scale variations in gap size or mechanical runout in the cone-plate region may have also contributed to the observed variability.

In the context of macroscopic blood flow through blood-contacting medical devices, other factors such as device variability, geometric uncertainties, blood handling, operator technique, and circuit variability contribute to experimental variability, making it challenging to determine whether these findings are directly translatable to such experiments. Nonetheless, the experimental setup in this study minimized all other sources of variation, making it possible to isolate and quantify intra-donor variability under conditions of exceptional experimental control.

Our investigation into the influence of sample count on the precision of mean hemolysis estimates revealed that increased sample size substantially narrows confidence intervals. Two measurements yielded broad variability (±0.3% HI around the total mean), but just five measurements reduced this uncertainty to ±0.2% HI. As shown in Figure 2, the combined mean HI of all donors was 0.55% HI, thus only performing two measurements might over- or underestimate the true HI by up to 60%. From an experimental standpoint, this challenges the conventional practice of using only a few replicates (often triplicates), which may underrepresent the true variability. From a modeling perspective, this underscores the importance of incorporating uncertainty quantification measures to represent the full distribution of possible outcomes, rather than relying solely on mean values.

These findings align with growing regulatory expectations from bodies such as the FDA, which emphasize verification, validation, and uncertainty quantification (VVUQ) as essential components of robust computational models for medical devices. Since most numerical hemolysis models currently rely on empirical relationships (e.g., Power Law models), the quality and representativeness of the underlying experimental data are critical. Existing literature often attributes differences in reported hemolysis values to factors such as species disparities [15] or device-specific designs and consequently different blood flow conditions [37]. Our results indicate that even under fixed conditions (single species, controlled shear stress, and exposure time), intra-donor variability can be substantial. This suggests that some of the discrepancies across studies may stem not only from differences in species or device designs but also from insufficient replication at given operating points. Supporting this, preliminary experiments using porcine blood at varying exposure times (Supplementary Figure S1) indicate that intra-donor variability is not confined to our single shear condition but persists across different flow regimes. Relying on as few as three samples to represent a single condition, as is common in many published studies, may lead to considerable uncertainty and potentially misleading model calibration. This finding is not only relevant for numerical hemolysis calibration but also potentially applicable to a wide range of experimental in vitro investigations involving blood, as previously demonstrated in the context of material hemocompatibility with an intra-variability of 40% CV [38].

By highlighting this inherent variability, our findings encourage a re-evaluation of current experimental protocols. Instead of spreading limited resources across numerous operating points with minimal replication, it may be more beneficial to invest in deeper replication at key conditions. Such an approach can uncover variability patterns that would remain hidden in sparser datasets. In particular, relying on a small number of replicates, such as the common practice of using triplicates, may underestimate the true uncertainty due to high intra-donor variability. For example, mechanistic studies or efforts to reduce technical noise may benefit from more replicates per donor, while studies aiming to capture population-level trends or stratify donor responses may prioritize a broader donor base. Our results aim to support experimentalists in making informed trade-offs between the number of donors and the number of replicates per donor, depending on the specific aims of their study.

Furthermore, incorporating variability directly into hemolysis models, rather than relying on a single mean curve, would represent a substantial step toward developing uncertainty-aware in silico predictions. These more comprehensive models could assist device developers and regulators in better understanding the range of plausible hemolysis outcomes, ultimately informing safer and more reliable device designs.

Despite these advances, some limitations must be acknowledged. First, our study focused on a single shear stress and exposure time condition using a single device setup and blood handling procedure. While this allowed for strict control and consistency, the applicability of our findings to other shear rates, exposure times, sample volumes, and device configurations remains to be confirmed. Second, our analysis focused primarily on hemolysis outcomes without delving deeply into underlying cellular or biochemical mechanisms. Future research might benefit from coupling extensive replication with more detailed cellular analyses or advanced imaging techniques to pinpoint the exact sources of intra-donor variability at the cellular or molecular level.

In summary, this study demonstrates that intra-donor variability is a substantial and previously underappreciated factor in hemolysis measurements. By showing how sample size can influence the precision of mean values, we emphasize the need for both increased experimental replication and robust uncertainty quantification in hemolysis modeling. Integrating these insights into future experimental designs and computational models will enhance the reliability of hemolysis predictions, facilitating more robust device development and regulatory approval processes for blood-contacting medical devices.
